# Mutual information reveals multiple structural relaxation mechanisms in a model glass former

**DOI:** 10.1038/ncomms7089

**Published:** 2015-01-22

**Authors:** Andrew J. Dunleavy, Karoline Wiesner, Ryoichi Yamamoto, C. Patrick Royall

**Affiliations:** 1 H.H. Wills Physics Laboratory, Tyndall Avenue, Bristol BS8 1TL, UK; 2 School of Chemistry, Cantock’s Close, University of Bristol, Bristol BS8 1TS, UK; 3 Bristol Centre for Complexity Sciences, Bristol BS8 1TW, UK; 4 School of Mathematics, University of Bristol, Bristol BS8 1TW, UK; 5 Department of Chemical Engineering, Kyoto University, Kyoto 615-8510, Japan; 6 Centre for Nanoscience and Quantum Information, Tyndall Avenue, Bristol BS8 1FD, UK

## Abstract

Among the key challenges to our understanding of solidification in the glass transition is that it is accompanied by little apparent change in structure. Recently, geometric motifs have been identified in glassy liquids, but a causal link between these motifs and solidification remains elusive. One ‘smoking gun’ for such a link would be identical scaling of structural and dynamic lengthscales on approaching the glass transition, but this is highly controversial. Here we introduce an information theoretic approach to determine correlations in displacement for particle relaxation encoded in the initial configuration of a glass-forming liquid. We uncover two populations of particles, one inclined to relax quickly, the other slowly. Each population is correlated with local density and geometric motifs. Our analysis further reveals a dynamic lengthscale similar to that associated with structural properties, which may resolve the discrepancy between structural and dynamic lengthscales.

## Abstract (web summary)

One mystery of glass transition from supercooled liquid is the lack of apparent change in structure, which is in contrast to a large change in dynamics. Here Dunleavy *et al*. provide a possible solution to this discrepancy by simulating dynamic correlation using a mutual information approach.

## Graphical abstract

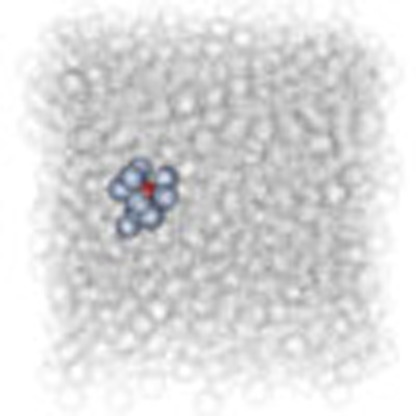

The nature of the glass transition has proved a long-standing challenge in condensed matter. Whether there is a true thermodynamic transition at finite temperature or whether the structural relaxation time in glass-forming liquids diverges only at zero temperature remains controversial. Furthermore, the solidification that is manifested in the glass transition without any significant change in structure assumed by the constituent particles challenges the concept that structure should somehow underlie dynamics. An understanding of this solidification process is crucial in key emerging technologies. Metallic glass, for example, exhibits superior mechanical properties compared with other materials, but exploitation is limited because large parts cannot be fabricated. A second example is chalcogenide glassformers that are on the brink of commercialization in next-generation non-volatile memory^1^.

Among the key developments in our understanding of the glass transition in recent years is dynamical heterogeneity. Glassy supercooled liquids do not relax uniformly but exhibit fast and slow regions with an associated dynamic lengthscale which grows on deeper supercooling^1^,^2^. Dynamical heterogeneity has various interpretations: it may be due to the formation of ‘cooperatively rearranging regions’^3^, which undergo entropic melting (random first-order transition (RFOT) theory^3^,^4^); the hierarchical interactions of mobility excitations^5^; or the presence of geometric motifs such as icosahedra^6^.

Some of these approaches^3^,^4^,^6^ anticipate an increasing dynamic lengthscale that is accompanied by a rise in a structural lengthscale. Furthermore, the super-Arrhenius increase in relaxation times exhibited by many glassformers implies an increasing structural lengthscale at sufficient supercooling^7^. Many attempts have been made to find coincident increases in dynamic and structural lengthscales^2^, but with mixed results: some find identical scaling between a dynamic correlation length *ξ*_4_ fitted to a four-point spatiotemporal ‘dynamic structure factor’^8^,^9^ in experiment^10^ and computer simulation^11^,^12^,^13^. However, others find that while *ξ*_4_ increases strongly, structural correlation lengths grow weakly if at all^14^,^15^,^16^,^17^,^18^,^19^. A significant unresolved question^20^ is why the four-point dynamic correlation length *ξ*_4_ (refs 9, 19) grows to around five to ten particle diameters^12^,^19^ in the regime accessible to simulation (around five decades of increase in relaxation time relative to a normal liquid), while values for the dynamic correlation length obtained from indirect measurements around the experimental glass transition (some 14 decades of increase in relaxation time) also grow to around five to ten diameters^21^. The limit of the regime accessible to simulation roughly coincides with the mode-coupling transition at which divergence of *ξ*_4_ is suggested from some simulation data^8^,^19^; however, deeper quenching indicates a crossover in *ξ*_4_ behaviour around the transition^22^.

This suggests at least two possibilities: first that the dynamic length does not change significantly in the nine decades of relaxation time between the simulation regime and the experimental glass transition and indeed definitions other than *ξ*_4_ exhibit non-monotonic behaviour around the mode-coupling transition, decreasing on deeper supercooling^16^. Second that dynamic correlations of the kind envisioned in, for example, cooperatively rearranging regions^3^,^4^ might in fact correspond to a different lengthscale to that of *ξ*_4_. This brings us to consider exactly what a dynamic lengthscale might measure. Cooperative relaxation (correlation in particle motion) is an important quantity, and has been identified with string-like motion^23^,^24^ and dynamic facilitation^5^. String-like motion implies mobile regions with a fractal dimension <3, which may be understood within Adam–Gibbs^24^ and RFOT theory^25^ at the limited degree of supercooling accessible to simulation. RFOT theory predicts more compact mobile regions at deeper supercooling^25^.

Here we use mutual information to identify the correlated propensity, which underlies that dynamical heterogeneity is encoded in the structure. We consider a glass former in the isoconfigurational ensemble in which the system is simulated many times from one equilibrium configuration but with randomized dynamics: at the start time, *t*=0, the particles in each simulation are identically positioned; as *t* increases, the trajectories of the simulations diverge^26^. Here the propensity is the mean displacement of the particles averaged across the isoconfigurational ensemble. Thus far, limited connection between structure and dynamical properties in the isoconfigurational ensemble has been found^26^,^27^,^28^,^29^, with the notable exception of normal modes, which are correlated with relaxation at very short times^30^ and larger ‘avalanche’ events on longer timescales^31^. The isoconfigurational ensemble allows us to measure correlations in particle dynamics that are encoded in the initial configuration of the system. By determining the mutual information in the displacement probability distributions of pairs of particles, we find all pairwise correlations in the propensity. Our analysis reveals two modes of correlated motion: ‘early’ correlations that involve mobile particles and ‘late’ correlations between particles that remain immobile until long after the structural relaxation time *τ*_*α*_. These two populations are associated with distinct geometric motifs and local density. We further identify the lengthscale of dynamic correlations found by our method whose magnitude is similar to correlation lengths based on structural quantities.

## Results

### Correlated particles reveal two modes of relaxation

**Figure 1:**
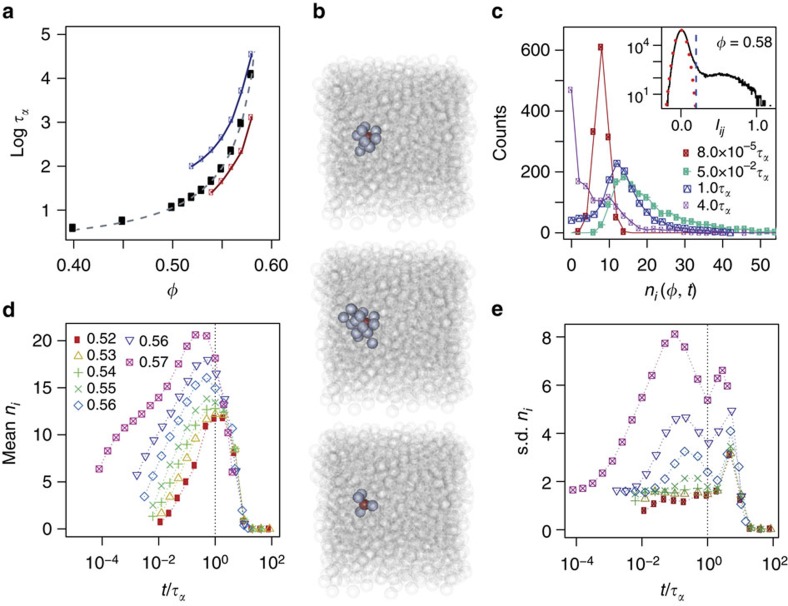
Identifying correlated partners. (**a**) ‘Angell’ plot of relaxation time as a function of *φ* (black symbols) fitted with a Vogel–Fulcher–Tamman form as defined in the text. Red data points are early peaks in **e** and blue data points are late peaks in **e**. (**b**) Examples of particles (red) and their significantly correlated partners (blue) in a system with *φ*=0.58 at 0.01*τ*_*α*_, 0.125*τ*_*α*_ and 8*τ*_*α*_ from top to bottom. Correlated partners are typically close together. The particles are rendered actual size. (**c**) The distribution of *n*_*i*_ (the number of significantly correlated partners, see text) at selected times with *φ*=0.58. The inset shows a characteristic histogram of mutual information between particle displacements. The Gaussian distribution (red dotted line) models the noise in the mutual information estimates. A threshold is used (dashed vertical line) to define significantly correlated particle pairs. (**d**) The time evolution of the mean of the distribution of *n*_*i*_(*t*) for *φ* between 0.52 and 0.58. (**e**) The standard deviation of the distribution of correlated partners *n*_*i*_(*t*) for *φ* between 0.52 and 0.58. Note the growth of an early peak as the volume fraction is increased.

Our analysis is based on isoconfigurational simulations of a five-component system of hard spheres (see Methods) over a range of volume fractions *φ*, which undergoes a glass transition at *φ*_0_≈0.608 according to the Vogel–Fulcher–Tamman fit *τ*_*α*_(*φ*)=*τ*_0_exp[*A*/(*φ*_0_−*φ*)] as shown in Fig. 1a. Here *τ*_0_ is a timescale in the normal liquid and *A* is a constant related to the fragility^1^,^19^. Our system shows no sign of crystallization. We quote length in units of the mean diameter and timescales in simulation time units. In an isoconfigurational ensemble, the displacement of the *i*th particle at a given time is a random variable *X*_*i*_(*t*). The randomness of such a variable can be quantified by the Shannon entropy 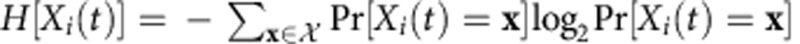 where 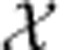 is the set of possible values of *X*_*i*_(*t*). The difference between the Shannon entropy of two random variables and the sum of the individual entropies is a measure of correlation between those variables, the mutual information: *I*[*X*_*i*_(*t*);*X*_*j*_(*t*)]=*H*[*X*_*i*_(*t*)]+*H*[*X*_*j*_(*t*)]−*H*[*X*_*i*_(*t*),*X*_*j*_(*t*)] where *i*,*j* index a pair of particles (see Methods). By measuring the mutual information between these displacement distributions, we obtain a value for the strength of the correlation in displacement of each pair of particles in the system as a function of time. Here we consider vector displacements between particles. We threshold the mutual information values (see Fig. 1c) to define a set of significantly correlated pairs of particles. For reasonable changes of the threshold value, our results are unaffected: see Supplementary Fig. 1. We define *n*_*i*_(*t*) as the number of particles whose position is significantly correlated with that of particle *i* at time *t* and *τ*_*α*_ the structural relaxation time as the time at which the intermediate scattering function *F*(*k*,*t*=*τ*_*α*_)=1/*e* (see Methods). For *φ*=0.58, *n*_*i*_(*t*) as a function of time is shown in Fig. 1c (other state points are shown in Supplementary Fig. 2). The value of *n*_*i*_(*t*) can be considered a proxy for the extent of collective motion that particle *i* is engaged in. As one can see in Fig. 1b, the correlated partners of a given particle tend to be arranged locally and in a reasonably compact manner. Whether these regions indeed become more compact at deeper supercooling as predicted^25^ is worthy of investigation, but would require large amounts of computer time.

Figure 1d shows the mean *n*_*i*_(*t*) (averaged over the particles) for different *φ* and *t*. As *φ* becomes larger the mean increases, with a maximum at *t*~*τ*_*α*_ for low *φ* and at *t*<*τ*_*α*_ for high *φ.* The system of highest volume fraction demonstrates increased correlations at ‘early’ times (*t*≪*τ*_*α*_). This indicates that the relaxation dynamics are changing qualitatively with volume fraction. This change in relaxation mechanism is also seen in the standard deviation of the *n*_*i*_(*t*) distribution (Fig. 1e). At low densities, there is a single peak in the s.d. at long times, but as the volume fraction is increased an earlier peak grows.

**Figure 2:**
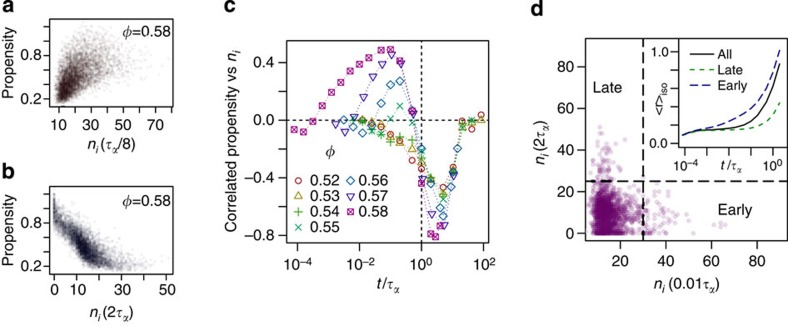
Characteristics of fast and slow particle populations. (**a**) Positive correlation between propensity and *n*_*i*_ at a short time of *τ*_*α*_/8. (**b**) Negative correlation between propensity and *n*_*i*_ at a long time of 2 *τ*_*α*_. (**c**) The correlation between *n*_*i*_(*t*) and propensity (the expected displacement after *t*=*τ*_*α*_). For all densities, *n*_*i*_ is anticorrelated with propensity at *t*≥*τ*_*α*_ indicating the existence of stable structures in the system. An early peak in correlation grows with volume fraction mirroring the importance of early collective relaxation in dense systems. (**d**) Two separate populations of early and late correlated particles for *φ*=0.58. There are no particles in the top right quadrant. The inset shows the expected displacement of the groups over time.

These two peaks motivate us to make a distinction between particles with high *n*_*i*_(*t*) at the time of the earlier peak (‘early’ times) and those with the later peak *n*_*i*_(*t*) at *t*>*τ*_*α*_ (‘late’ times). This distinction has dynamical implications. Figures 2a and b show correlations between propensity and *n*_*i*_ at short (positive correlation) and long (negative correlation) times, respectively. Figure 2c shows the Pearson correlation coefficient between *n*_*i*_(*t*) and particle propensity. The propensity is determined by the displacement of the particle in each trajectory and averaged across the isoconfigurational ensemble. High *n*_*i*_(*t*) at late times is anticorrelated with propensity and this effect increases in magnitude with increasing *φ*. Conversely, at early times and high *φ*, *n*_*i*_(*t*) is positively correlated with propensity. This positive correlation grows with *φ* in a similar manner to the early peak in the variance of *n*_*i*_(*t*): we take this as further evidence that there is a change in dynamical behaviour as *φ* is increased. Below we enquire as to the structural characteristics of these early- and late-moving regions.

We note that there are no particles at any *φ* that have high *n*_*i*_(*t*) at early and late times. Figure 2d shows the joint distribution of early and late *n*_*i*_(*t*) for a high density system (*φ*=0.58). We are able to divide the particles into early (high propensity), late (low propensity) and ‘normal’ populations on the basis of the *n*_*i*_(*t*) measurements. The presence of high *n*_*i*_(*t*) particles (which are also immobile) at *t*>*τ*_*α*_ indicates the presence of stable structures in the initial configuration that have long lasting influence over the dynamics. The distribution of points in Fig. 2d shows the relative populations of particles in these two modes of relaxation.

Distinct populations of particles are of course the hallmark of dynamic heterogeneity. Recently, a population of particles with a timescale shorter than *τ*_*α*_ has been related to diffusion, which decouples from full structural relaxation (*τ*_*α*_) in the Stokes–Einstein breakdown^24^. A second population of particles in immobile clusters has also been identified, which relaxed on timescales around *τ*_*α*_. We expect that our early-moving population may be related to such diffusive behaviour, while late-moving particles may correspond to the second (immobile) population in ref. 24. In Fig. 1a, we show the relaxation times of the fast and slow populations corresponding to the peaks in the s.d. of *n*_*i*_ in Fig. 1e. Like diffusivity^24^, the timescale of the fast population departs from that of structural relaxation, while the slow population shows some signs of approaching *τ*_*α*_ at deep supercooling.

### Correlations with structure

**Figure 3:**
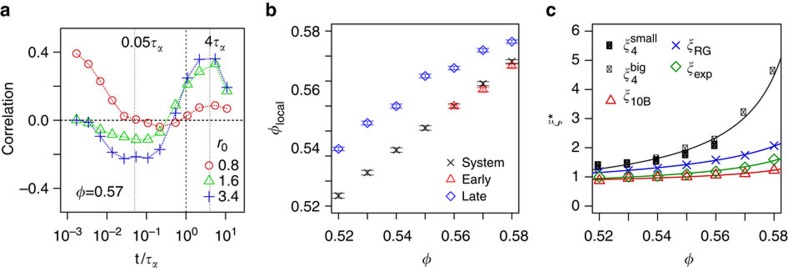
Local density and lengthscales in the system. (**a**) Pearson correlation between local volume fraction and *n*_*i*_(*t*) for *φ*=0.57. The local volume fraction of a particle *i* is the fraction of the volume of a sphere centred on particle *i* that is occupied by particles. The radius of the sphere is *r*_0_ times the diameter of particle *i*. (**b**) Average local volume fraction *φ*_local_ of early and late-relaxing particles and the whole system for various global volume fractions *φ*. Note that only the higher density systems (*φ*>0.55) have collectively moving early-relaxing particles. *φ*_local_ is calculated with *r*_0_=1.4, other values of *r*_0_ give similar results. Error bars are twice the standard error. (**c**) Structural and dynamic correlation lengths as a function of *φ*. Dynamical lengths *ξ*_4_ are calculated in conventional microcanonical simulations of different sizes as described in the text. *ξ*_exp_ and *ξ*_RG_ are dynamic lengthscales of significantly correlated particles in the isoconfigurational ensemble. *ξ*_10B_ is a structural lengthscale based on 10B clusters. Lines are a guide to the eye. 
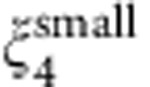
 and 
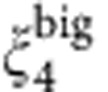
 correspond to the four-point correlation length from conventional simulations for different system sizes of *N*=1,372 and *N*=10,976 respectively.

Since the correlations discussed here are measured in the isoconfigurational ensemble, we know that they have a structural origin. They must be caused by the initial particle configuration as this is the only thing in common between the different trajectories^26^. To investigate structural features relevant to the collective motion, we consider two measures of local structure. The first of these is the local volume fraction (*φ*_local_) around a particle, which we define in a sphere of radius *r*_0_ centred on the particle. The local volume fraction has a pronounced effect on the correlated motion of the system. Figure 3a shows Pearson correlation coefficients between *φ*_local_ and *n*_*i*_(*t*) for a high-volume fraction system *φ*=0.57 (other state points are shown in Supplementary Fig. 3).

The effect of *φ*_local_ depends on the radius *r*_0_ and we identify a regime at very short times in addition to those so far discussed. For *r*_0_ less than a particle diameter, we find a positive correlation between high local volume fraction and *n*_*i*_(*t*) at very short time. Particles with high *φ*_local_ at very small *r*_0_ will be nearly in contact with one or more neighbours. This does not guarantee that the wider locality is particularly dense, but the potential for very early collisions means that such particles have high *n*_*i*_(*t*) at very early times. For all *φ*, these correlations are strongest before the ‘early’ time period when *n*_*i*_(*t*) and propensity are correlated at high *φ* (see Fig. 2). When *r*_0_ is increased beyond a particle diameter, there is a negative correlation between *φ*_local_ and *n*_*i*_(*t*) at early times and a somewhat stronger positive correlation at ‘late’ times (when *n*_*i*_(*t*) and propensity are anticorrelated). Since the particle displacements become correlated with each other through collisions, at the very earliest times the particles in the highest density regions of the system have more correlated partners than average (as they have had more opportunities to collide with other particles). However, in terms of the early and late populations highlighted in Fig. 2d, the former are more likely to be situated in regions of lower local volume fraction whereas the late particles are in regions of higher local volume fraction. Typically, the late-moving particles have mean *φ*_local_ 0.01–0.02 higher than the whole system average *φ*_local_. Figure 3b shows the mean *φ*_local_ for early, late and all particles for a range of *φ*. All lengthscales over which the correlated motion is measured are larger than typical displacements. For the *φ*=0.57 data shown in Fig. 3a, the displacements are 0.23(0.15) and 0.44(0.71) at *t*=0.05*τ*_*α*_ and 4*τ*_*α*_ for the early/fast and late/slow populations, respectively. Here the data in brackets are mean displacements for the whole system at each time. Displacements of the different populations at times relative to *τ*_*α*_ as a function of volume fraction are given in Supplementary Fig. 4.

More remarkable is the correlation of *φ*_local_ and *n*_*i*_(*t*) with larger *r*_0_ (the results for *r*_0_=1.6 and *r*_0_=3.4 are qualitatively similar). Here there is little correlation for low global volume fraction systems (*φ*<0.56, see Supplementary Fig. 3) but as *φ* is increased there is a notable positive correlation at *τ*_*α*_<*t*<10*τ*_*α*_ and anticorrelation at *t~*0.1*τ*_*α*_ shown in Fig. 3a. These two time periods correspond to those associated with the early- and late-relaxing particles. We find that late-relaxing particles are more likely to exist in denser parts of the system. This is consistent with the discussion below concerning the nature of the local structure of the fast and slow regions. The increase in *φ*_local_ associated with late-relaxing particles is more pronounced than the reduction associated with early-relaxing particles, see Fig. 3b. For example, for *φ*=0.57, the average *φ*_local_ (*r*_0_=1.4) for early- and late-relaxing particles (defined in the same way as the populations in Fig. 2d in the main text) are 0.566 and 0.582, respectively (compared with a system-wide average of 0.569). Supplementary Fig. 5 shows plots of *φ*_local_ versus *n*_*i*_(*t*) for *φ*=0.57 at *t*=0.1*τ*_*α*_ and *t*=2*τ*_*α*_. For *φ*=0.58, the equivalent figures are 0.576 and 0.586 for early and late particles compared with a system average of 0.578.

**Figure 4:**
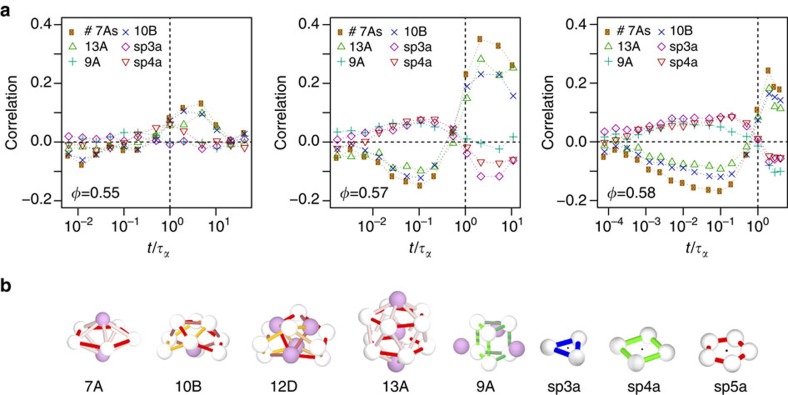
Local structure and dynamic populations. (**a**) Correlation between membership of topological clusters and *n*_*i*_(*t*) for volume fraction (left to right) *φ*=0.55, 0.57 and 0.58. #7A is the number of 7A clusters a particle participates in; for the other clusters we measure only whether the particle participates in that cluster or not. (**b**) A selection of the stable clusters (7A, 10B, 12D, 13A); the unstable cluster 9A; and the shortest-path rings (sp3a, sp4a, sp5a): the black dot indicates the central spindle axis to which particles may be added.

Our second measure of local structure is the topological cluster classification that is based on the bond network between particles defined through a Voronoi decomposition (see Methods). This identifies geometric motifs whose bond network is identical to certain clusters. Particular clusters are known to be long-lived in hard sphere systems^19^. For example, particles that participate in 10B and 13A (icosahedra) have higher *n*_*i*_(*t*) at *t*≥*τ*_*α*_ and (at high densities) low *n*_*i*_(*t*) at *t*≪*τ*_*α*_ indicating that these clusters are correlated with stability at long times >*τ*_*α*_ (see Fig. 4). These stable clusters are geometrically related to each other: they are all subclusters of 13A icosahedra or 12D (the 12D cluster exists where there are interlocking 13A clusters), and all contain three- and five-membered shortest-path rings but no four-membered rings. These more stable clusters are notable for incorporating a number of pentagonal bipyramid 7A clusters (a five-membered ring with two neighbours). Since the particles can be part of more than one cluster, we also show the number of pentagonal bipyramids a particle is in which we express as #7A.

There are also clusters that are correlated with particles with high *n*_*i*_(*t*) at early times (and thus with high propensity). The largest is the 9A cluster, which is based on a triangular prism and includes three four-membered rings but no pentagonal bipyramid clusters. Other clusters that are correlated with early (fast) particles include rings of 3, 4 and 5 particles (sp3a, sp4a and sp5a clusters, respectively), which have no adjacent ‘spindle’ particles bonded to all members of the ring. These clusters may be thought of as ‘missing’ a bond, which may explain the reduced local volume fraction associated with ‘fast’ particles. By contrast, pentagonal bipyramids that optimize local packing are correlated with more immobile regions.

### Dynamic and static lengthscales

Our approach provides a means to investigate the lengthscales of dynamic correlations and directly compare them with other lengthscales such as those associated with structural motifs and the four-point dynamic correlation length *ξ*_4_. In Fig. 3c, we compare the lengthscales of the correlated motion we measure in the isoconfigurational ensemble to *ξ*_4_, which is calculated from a (standard) simulation in the microcanonical ensemble (see Methods)^8^. We consider two system sizes to mitigate any finite-size effects^14^. Also shown is a structural lengthscale, *ξ*_10B_, which measures the size of domains of 10B clusters determined through a fit to the density–density correlation function of particles in 10B clusters (10B is associated with the population of slow particles)^19^. To obtain *ξ*_10B_, we fit a real-space Ornstein–Zernike (OZ) envelope to the pair-correlation function for particles in 10B clusters *g*_10B_(*r*): *g*_10B_(*r*)/*g*(*r*)~1/*r* exp[−*r*/*ξ*_10B_]. Further details are provided in the Methods.

We introduce two measures to determine the lengthscale of the correlated dynamics in the isoconfigurational ensemble: *ξ*_RG_ is the radius of gyration of each particle and its significantly correlated partners; *ξ*_exp_ characterizes the decay of mutual information with separation between significantly correlated pairs using an exponential fit. Both are determined at the peak value of the mean of *n*_*i*_(*t*) (~*τ*_*α*_ ) as shown in Supplementary Fig. 6. These dynamic lengthscales depend on a variable *c*_*ij*_(*t*) that is equal to 1 when particles *i* and *j* are significantly correlated and 0 otherwise. *ξ*_RG_ is calculated as the radius of gyration of particles that are significantly correlated with a central particle (this is averaged over all particles as the central particle). *ξ*_exp_ is calculated by plotting *c*_*ij*_(*t*) as a function of the distance between particles *i* and *j* and fitting an exponential with decay length *ξ*_exp_. In both cases, we take the maximum value (over *t*) as the representative for each *φ*. A full description of these lengthscales is given in the Methods.

The structural correlation length *ξ*_10B_ shows a modest increase on increasing volume fraction, comparable to that previously observed in a wide variety of systems using many different measures^2^,^14^,^15^,^16^,^17^,^18^,^19^. A similar behaviour is found for both dynamic correlation lengths, *ξ*_RG_ and *ξ*_exp_. However, our results do not match the rapid growth of the four-point dynamic correlation length *ξ*_4_. Indeed, while the generic behaviour of *n*_*i*_(*t*) (Fig. 1) is similar to the dynamic susceptibility *χ*_4_(*t*) (see Supplementary Fig. 7), a significant difference is found in that *n*_*i*_(*t*) is rather weakly dependent on *φ* compared with *χ*_4_(*t*).

Here we discuss the meaning of the difference between the measures of correlated dynamic motion we have introduced, *ξ*_RG_ and *ξ*_exp_, compared with *ξ*_4_. First we consider any consequences of working with the isoconfigurational ensemble which, alas, does not lend itself to measurements of an equivalent of *ξ*_4_ due to the lack of statistics. We therefore determine the dynamic susceptibility *χ*_4_, which we show in Supplementary Fig. 7. In the dynamical regime accessible to simulation, the isoconfigurational ensemble leads to a drop in *χ*_4_ relative to the microcanonical ensemble. In other models, *ξ*_4_ appears around a factor of 2 lower in the isoconfigurational ensemble^19^,^32^ and we see no reason to suppose our hard spheres would be significantly different^19^. Therefore, even within the isoconfigurational ensemble, *ξ*_4_ is expected to be rather larger than the dynamic length we measure. We thus conclude that our findings should not, in a qualitative sense, depend on the ensemble. Instead, the reason our dynamical lengths are smaller than *ξ*_4_ is that the correlated motion we measure considers whether particles influence the behaviour of one another and the range over which this occurs. In other words, we measure cooperative motion of the kind envisioned in the Adam–Gibbs and RFOT theories^1^,^3^,^4^,^33^.

On the other hand, *ξ*_4_ measures how large the fast and slow regions tend to be in the system, and can be affected by the average distance between propensity excitations^5^ and potentially by coupling between different mobile regions^25^. These do not contribute to our measurements unless the motion is correlated throughout although it is worth noting that propensity excitations typically have the same lengthscale as *ξ*_RG_ and *ξ*_exp_. Alternatives to *ξ*_4_ have also been proposed, for example, defining a lengthscale by considering broken bonds rather than mobility, *ξ*_*b*_. A recent comparison with *ξ*_4_ indicates a similar behaviour between *ξ*_*b*_ and *ξ*_4_ (ref. 34). It is also possible to use quenched disorder to define a dynamic length^16^. Such lengths do not increase as markedly as *ξ*_4_. In fact, there is some decrease around the mode-coupling transition that could be related to more compact mobile regions at deeper supercooling^25^ and perhaps to structural lengthscales.

Since the structural length *ξ*_10B_ behaves similarly to the lengthscales of collective motion *ξ*_RG_ and *ξ*_exp_, we suggest that a link between structure and dynamics may be identified through our method. Therefore, one possible resolution of the disparity between structural and dynamic lengthscales identified previously^2^,^15^,^16^,^17^,^18^,^19^ is to consider dynamic lengthscales, which measure the correlated motion. Here we have considered one model that has specific local structures. However, in other models, although the local structure is different, it has been shown^15^,^18^,^19^ that the structural correlation lengthscales in a similar manner. Even if the local structure may be hard to define (or not yet known), we note that order-agnostic methods give similar behaviour for the structural correlation lengths as we have considered here^15^,^17^. In 2D^12^,^35^ and where there can be local hexagonal order^11^,^12^, the structural correlation length can be larger. Our analysis, therefore, pertains to model systems with relatively high glass-forming ability, and, for example, to metallic glassformers that exhibit similar structural behaviour^36^. We find it tempting to imagine that a correlation length related to the glass transition increases rather more slowly than *ξ*_4_ in a manner consistent with structural lengths and the dynamic lengthscales we have measured.

## Discussion

By computing the mutual information between the vector displacements of particles in an isoconfigurational ensemble of polydisperse hard spheres, we have been able to identify correlations between structure and dynamics that until now have escaped detection. The initial configuration in the system influences relaxation by predisposing particular particles to collective motion^26^. In particular, encoded in the initial structure is a population of particles with low propensity that undergoes highly correlated motion at times longer than the structural relaxation time *τ*_*α*_. These slow particles are found in regions of high local density and are associated with geometric motifs based on pentagonal bipyramids. Approaching the glass transition as the system becomes more dense, collective rearrangement becomes more important to early relaxation and we find groups of dynamically correlated early-moving fast particles. These fast particles are associated with distinct, less-stable structural motifs with lower local density, and we expect that these are also correlated with low-frequency ‘soft’ normal modes^30^. Our results are consistent with a change in dynamical behaviour towards relaxation via cooperatively rearranging regions^3^,^4^. However, one could also consider the early correlations we find as propensity excitations^5^.

We offer a resolution to the conundrum concerning structural and dynamic lengthscales based on the four-point length *ξ*_4_: considering correlated motion in the isoconfigurational ensemble provides a means to identify a dynamic length attributed to cooperative rearrangements. This lengthscale is similar in magnitude and increases weakly with supercooling in a similar way to lengthscales based on structural measures. We recall that the rate of increase of the four-point length *ξ*_4_ with supercooling (see Fig. 3c) in the regime accessible to computer simulation is sufficiently rapid as to be inconsistent with dynamic lengthscales inferred at the molecular glass transition^2^,^20^,^21^. It is possible that such a rapid increase in dynamic lengthscale as that exhibited by *ξ*_4_ is not sustained on deeper supercooling^16^,^22^. Conversely, the dynamic correlation lengths we have identified are similar to those found directly from structural quantities and thus may increase continuously on supercooling towards the molecular glass transition. We hope that our work will stimulate the development of other measures of dynamic lengthscales focussing on correlated motion and will lead to a consensus of similar scaling of structural and dynamical lengthscales.

## Methods

### Isoconfigurational ensemble

The simulations were carried out using a polydisperse mixture of 1,372 hard spheres of equal mass but with different relative diameters (0.799*σ*, 0.861*σ*, 0.899*σ*, 0.938*σ* and *σ*)^19^. This mixture was evolved with event-driven molecular dynamics using the DynamO package^37^ for a range of volume fractions between *φ*=0.52 and *φ*=0.58. The relaxation time *τ*_*α*_ of the system was calculated from the self-intermediate scattering function 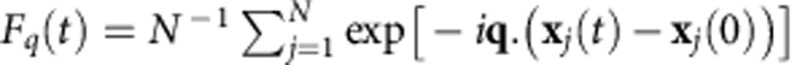 where *N* is the number of particles and **x**_*j*_(*t*) is the position of particle *j* at time *t*. We spherically averaged this expression over |**q**|=2*π*/*σ*. In the isoconfigurational ensemble, an (equilibrium) configuration was chosen for a set of 2,048 simulation runs. Each run was started with different random initial velocity coordinates (drawn from the Maxwell–Boltzmann distribution). Four isoconfigurational ensembles were simulated at each state point.

### Mutual information

The set of particle displacements {**r**_*i*_(*t*)} of the system in the isoconfigurational ensemble is a random variable *X*(*t*) with a probability density function (pdf) *f*_*X*(*t*)_({**r**_*i*_(*t*)}). We are interested in the displacements of individual particles: these are the random variables *X*_*i*_(*t*) (*i* indexes the particles). The probability density function (pdf) of one particle *f*_*Xi*(*t*)_(**r**_*i*_(*t*)) and the joint pdf of two particles *f*_*Xi*(*t*),*Xj*(*t*)_(**r**_*i*_(*t*),**r**_*j*_(*t*)) are marginal distributions of *f*_*X*(*t*)_({**r**_*i*_(*t*)}). The mutual information between two continuous random variables *X* and *Y* is defined as:


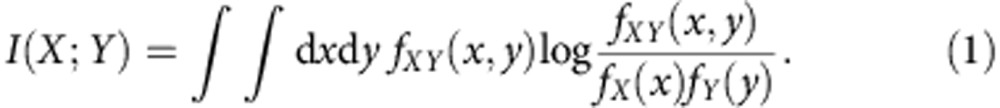


where *f*_*X*_(*x*) is the probability density function of the variable *X*. *I*(*X*;*Y*) is a non-negative function and measures the amount of correlation between *X* and *Y* (ref. 38).

The mutual information between the displacements of particles *i* and *j* at time *t* in a given isoconfigurational ensemble was quantified using the Kraskov–Stögbauer–Grassberger estimator^39^. We define two particles to be significantly correlated if their mutual information *I*_*ij*_(*t*) is greater than a threshold *I*_0_ whose value was chosen based on the noise floor of the mutual information measurements (see Fig. 1c inset). On the basis of this threshold, we denote the number of significantly correlated partners a particle *i* has at time *t* by *n*_*i*_(*t*). It is possible that the thresholding could introduce artefacts. However, our results are robust to large variations in the threshold value: changing the threshold merely rescales the *n*_*i*_(*t*) values, and the mean and s.d. of *n*_*i*_(*t*) at each time and state point. Supplementary Fig. 1 shows the effects of varying the mutual information threshold on the correlation between *n*_*i*_(*t*) and particle mobility (measured using the Pearson correlation coefficient). Here we set the threshold *I*_0_=0.2 nats. We see that the correlation values in change little except for *I*_0_≤0.12. At these values, the threshold is well below the noise threshold of our measurements.

### Lengthscales in the system

To obtain *ξ*_10B_, we fit an OZ envelope to the spatial correlations of the pair-correlation function of particles in 10B clusters.


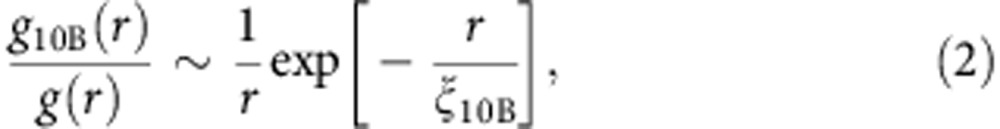


where


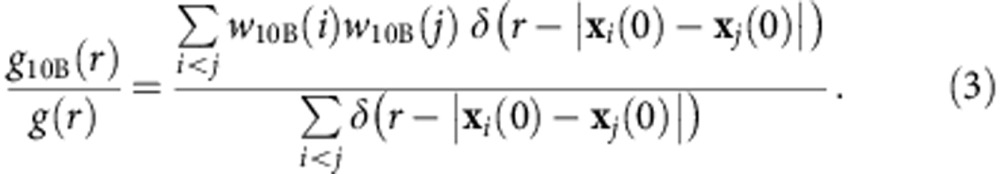


where *w*_10B_(*i*) equals 1 for particles in 10B clusters and zero otherwise.

To measure the four-point dynamic correlation length, *ξ*_4_ we calculate the dynamic susceptibility *χ*_4_(*t*) from the immobile particles in the system where


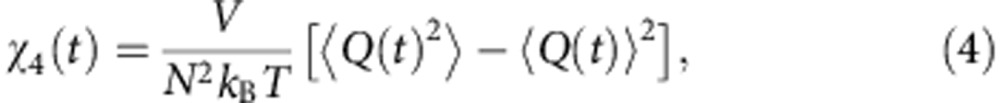



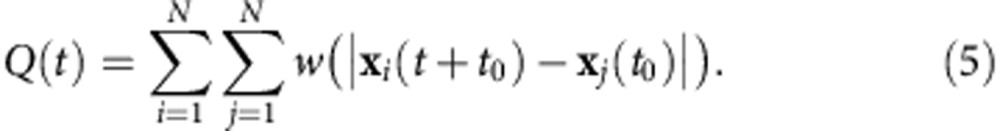


Here *i* and *j* index the particles and the overlap function *w*(|**x**_*i*_(*t*+*t*_0_)−**x**_*j*_(*t*_0_)|) is defined to be unity if |**x**_*j*_(*t*+*t*_0_)−**x**_*l*_(*t*_0_)| ≤*a*, 0 otherwise, where *a*=0.3. Supplementary Fig. 7 shows *χ*_4_(*t*) for various volume fractions. *χ*_4_(*t*) exhibits a peak at *t*=*τ*_*h*_, which corresponds to the timescale of maximal heterogeneity in the dynamics of the particles. We then construct the four-point dynamic structure factor *S*_4_(*k*,*t*):


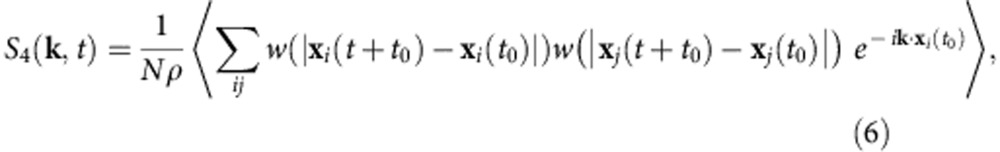


where *i* and *j* are particle indices and **k** is the wavevector. The four-point dynamic correlation length *ξ*_4_ was then obtained by fitting an OZ function to the spherically averaged *S*_4_(*k*,*τ*_*h*_) (ref. 8).

We calculate two dynamic lengthscales based on the mutual information. Let the variable *c*_*ij*_(*t*) be equal to 1 when particles *i* and *j* are significantly correlated and zero otherwise and we determine a radius of gyration at time *t* and volume fraction *φ* as


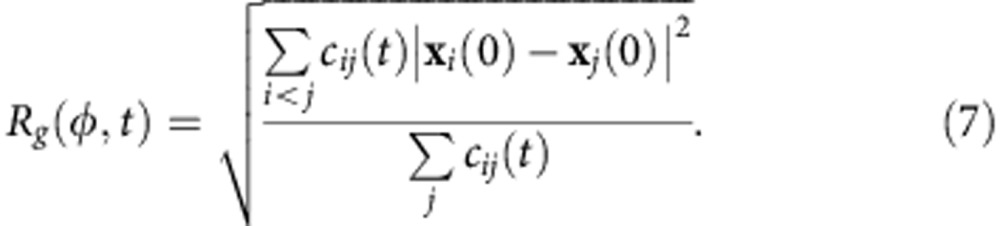


The second length we introduce, *ξ*_exp_, was calculated from the mutual information at time *t* and particle distance *r*,


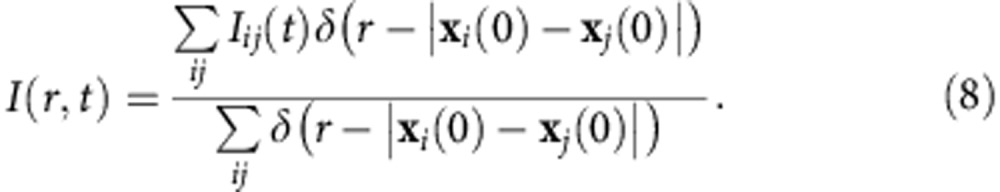


We fit an exponential function *I*(*r,t*)~exp[−r/*ξ*_exp_(*t*)] to define *ξ*_exp_.

Supplementary Fig. 6 shows both *R*_*g*_(*φ*,*t*) and *ξ*_exp_(*φ*,*t*) for various *φ*. To obtain a single length for a given *φ*, we follow the procedure for calculating the dynamical correlation length *ξ*_4_: we take the maximum value for each *φ*


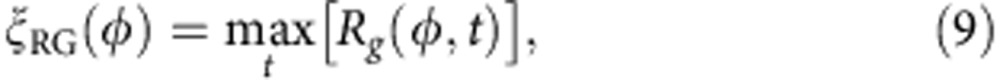



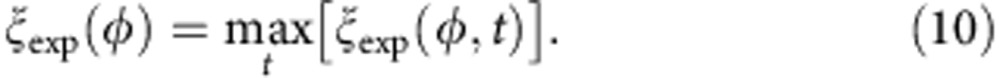


The value of *t* that maximizes these lengths generally coincides for a given *φ*. For *ξ*_exp_ with lower *φ*, the mutual information values are small enough (compared with the noise floor) to make exponential fitting unreliable. In these cases, we take *ξ*(*φ*) as the maximum value for the reliable fits and make sure that this value is at a time *t* close to that which maximizes *R*_*g*_(*φ*,*t*).

## Author contributions

A.J.D. performed the research. All authors designed the research, analysed data and wrote the manuscript.

## Additional information

**How to cite this article**: Dunleavy, A. J. *et al*. Mutual information reveals multiple structural relaxation mechanisms in a model glass former. *Nat. Commun.* 6:6089 doi: 10.1038/ncomms7089 (2015).

## Supplementary Material

Supplementary Information Supplementary Figures 1-7.
