# Supplementary material for: Mutual information reveals multiple structural relaxation mechanisms in a model glass former
Source: Nat Commun. 2015 Jan 22;6:6089. doi: 10.1038/ncomms7089 (PMC4354007; doi:10.1038/ncomms7089)
Supplement: Supplementary Information — Supplementary Figures 1-7. [file ncomms7089-s1.pdf]

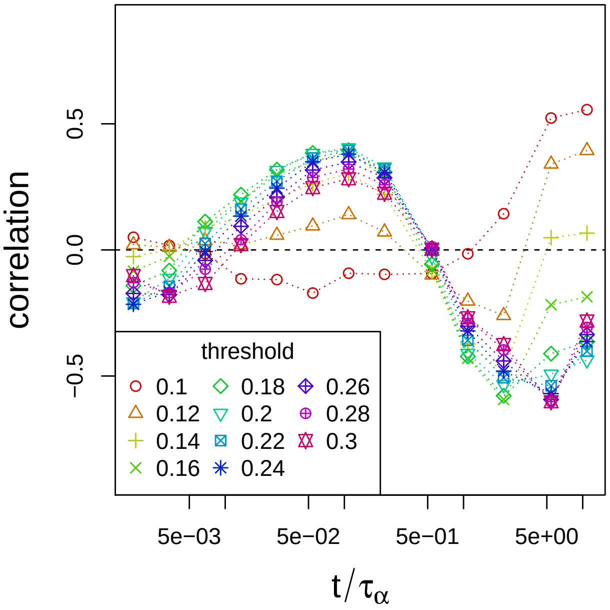

Supplementary figure 1: The effect of varying the mutual information threshold on the Pearson correlation coefficient between $n_i(t)$ and particle mobility. Here $\phi = 0.57$. This plot is representative of the effect of varying the threshold for all ϕ.

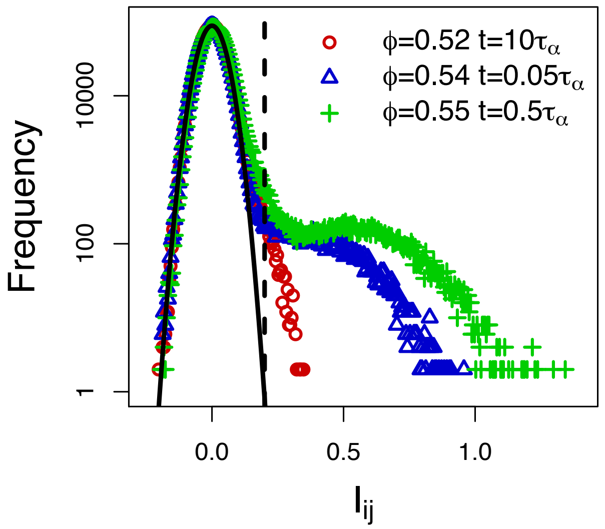

Supplementary figure 2: The distribution of n_i. n_i (the number of significantly correlated partners) is shown at selected times and volume fractions. The dashed line denotes the threshold we take and the solid line is a Gaussian fit to the distributions.

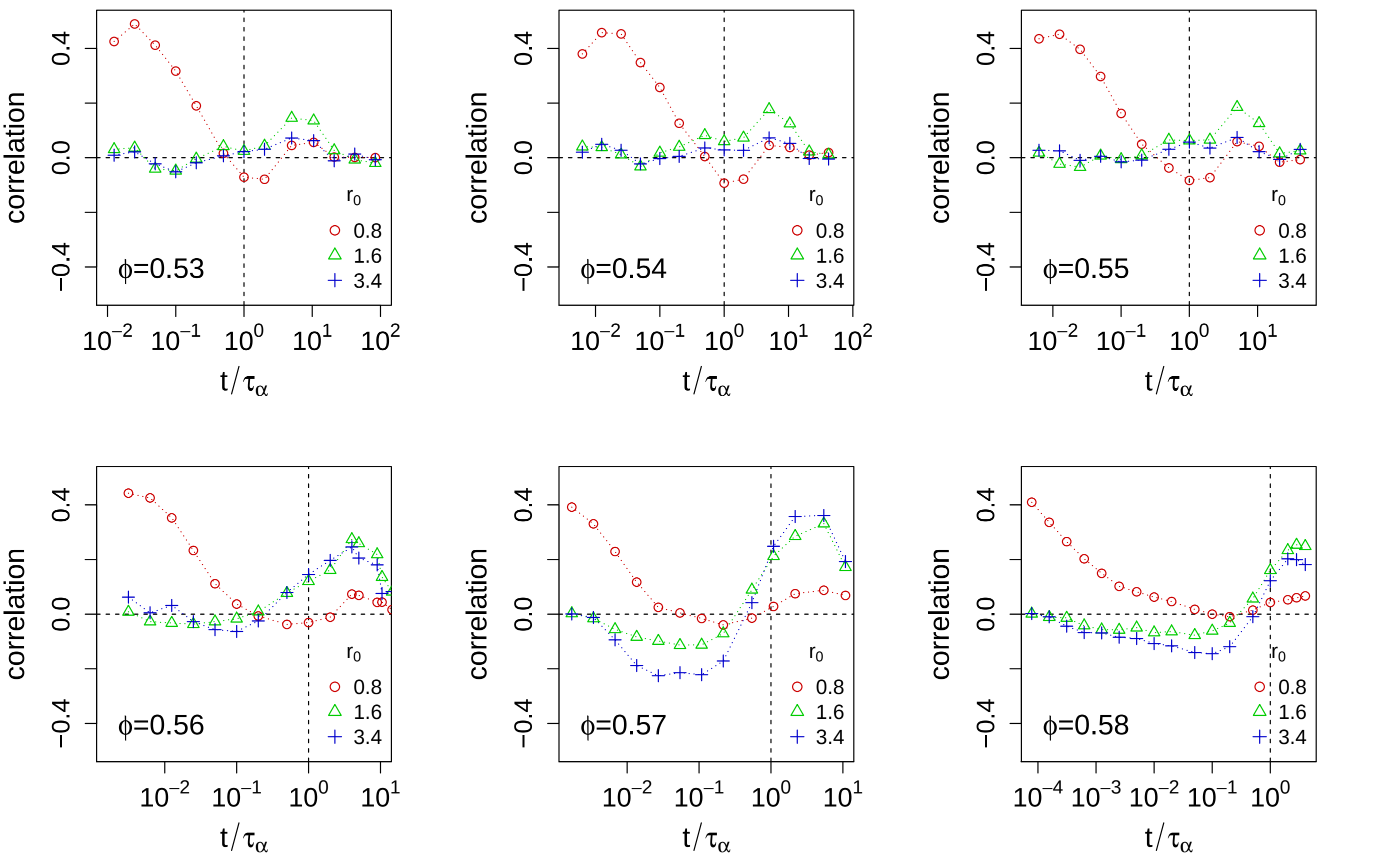

Supplementary figure 3: Pearson correlation between $\phi_{\rm local}$ and $n_i(t)$. $n_i(t)$ is the number of correlated partners particle i has after time t (as defined in the main text). The range of the local volume over which $\phi_{\rm local}$ is calculated can be varied. We show $\phi_{\rm local}$ calculated over spherical regions of radii $r_0 = 0.8$, 1.6 and 3.4 particle diameters. The correlation between $\phi_{\rm local}$ and $n_i(t)$ depends strongly on r_0. For all ϕ the correlation with $r_0 = 0.8$ is strongest at $t \ll \tau_\alpha$.

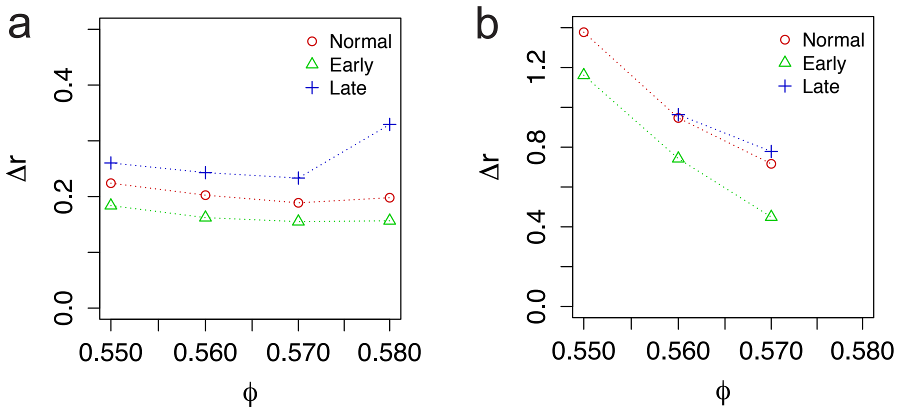

Supplementary figure 4: Displacements of the different populations as a function of volume fraction. "Normal" is the average displacement of all particles in the system, "early" corresponds to early (fast) moving particles and "late" is the average displacement of late (slow) moving particles. Data are shown for two times, $t = 0.05\tau_\alpha$ in (a) and $t = 4.0\tau_\alpha$ in (b).

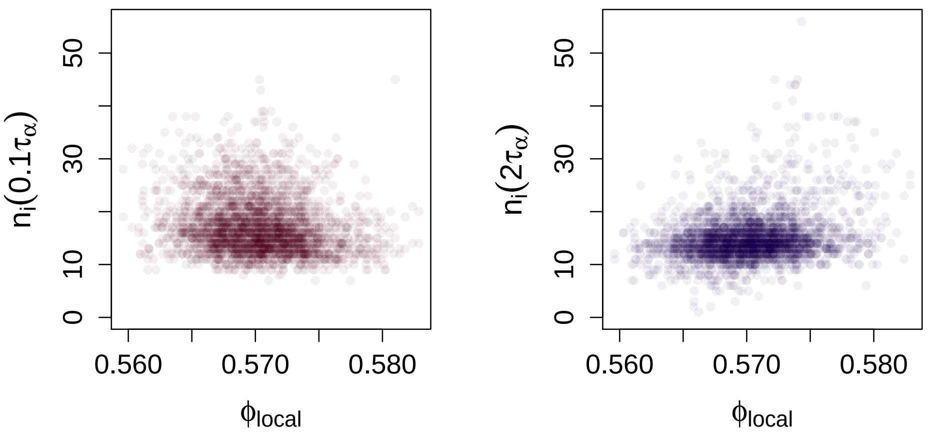

Supplementary figure 5: Plots of $\phi_{\rm local}$ versus $n_i(t)$ for $\phi = 0.57$ at $t = 0.1\tau_\alpha$ and $t = 2\tau_\alpha$. $\phi_{\rm local}$ is measured with radii $r_0 = 3.4\sigma$. These show that the correlations are a property of a small number of high $n_i(t)$ particles. Particles with low $n_i(t)$ exist at many $\phi_{\rm local}$. However, those with high $n_i(t)$ are more likely to take high (or low) $\phi_{\rm local}$ (depending on the value of t). The main effect is that the late relaxing particles (those with high $n_i(t)$ at $t > \tau_\alpha$) are positioned in regions of above average $\phi_{\rm local}$. Figure 3b in the main text shows the average $\phi_{\rm local}$ for early and late relaxing particles (defined using two values of $n_i(t)$ to partition the particles as in Fig. 2c in the main text). For all system volume fractions ϕ the late relaxing particles have a higher than average $\phi_{\rm local}$.

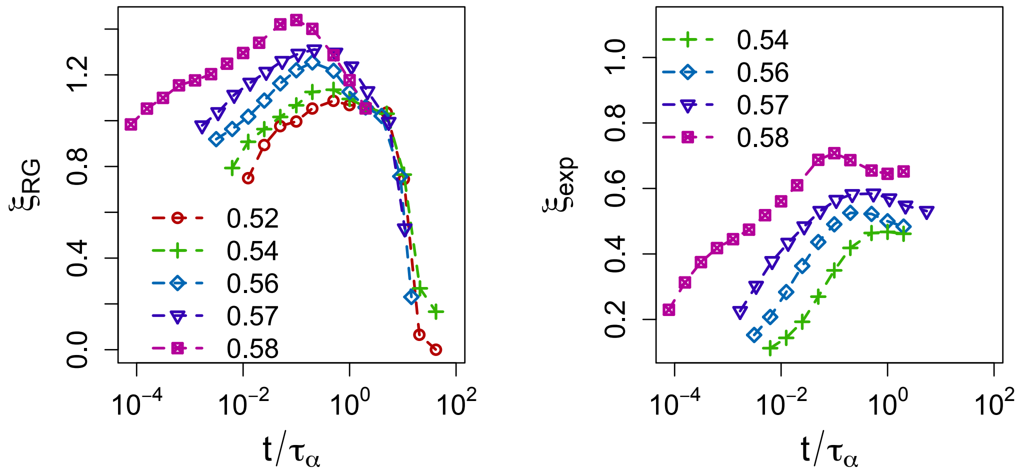

Supplementary figure 6: The two length-scales derived from our mutual information measurements.: $\xi_{\rm RG}$ is based on the radius of gyration of partner particles; and $\xi_{\rm exp}$ is based on an exponential fit to the decay of the pairwise mutual information (see Methods for further details).

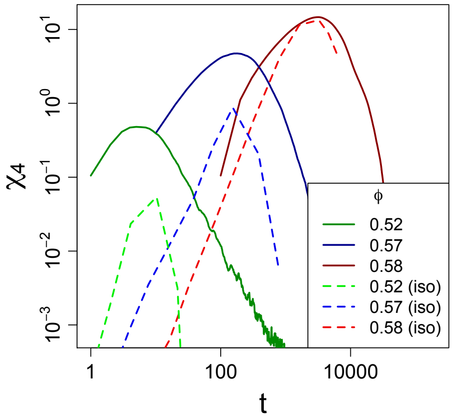

Supplementary figure 7: $\chi_4(t)$ **for the hard sphere system**. Solid lines are Eq. 4 at the volume fractions indicated i.e. they correspond to conventional simulations in the micro canonical ensemble. Dashed lines correspond to Eq. 4 where we have substituted the displacement with the propensity, i.e. i.e. the data are calculated from isoconfigurational ensemble simulations.
